# Effects of lateral hydrological connectivity on the relative abundance of water frogs (*Pelophylax* spp.) and common toads (*Bufo bufo*) using eDNA surveys

**DOI:** 10.1007/s10750-026-06110-5

**Published:** 2026-01-29

**Authors:** Boglárka Mészáros, Andrea Funk, Thomas Hein, Lukas Landler, Paul Meulenbroek, Didier Pont, Alice Valentini, Dénes Schmera, István Czeglédi, Tibor Erős

**Affiliations:** 1 HUN-REN Balaton Limnological Research Institute, Klebelsberg Kuno Street 3., Tihany, 8237 Hungary; 2 National Laboratory for Water Science and Water Security, HUN-REN Balaton Limnological Research Institute, Tihany, Hungary; 3 Christian Doppler Laboratory for Meta Ecosystem Dynamics in Riverine Landscapes, BOKU University, Vienna, Austria; 4 Institute of Zoology, BOKU University, Vienna, Austria; 5 Institute of Hydrobiology and Aquatic Ecosystem Management, BOKU University, Vienna, Austria; 6 SPYGEN, Savoie Technolac, Le Bourget du Lac, France

**Keywords:** Environmental DNA, Metabarcoding, Water frog, Common toad, Hydrology, Wetland

## Abstract

Riverine floodplains support diverse amphibian communities by providing essential habitats, with lateral hydrological connectivity (LHC or hydrology) determining local environmental conditions.

Our study evaluated the direct and indirect effects of LHC on the two most abundant species in a riverine floodplain. We assessed the relative abundance of water frogs (*Pelophylax* spp.) and common toads (*Bufo bufo*) using eDNA surveys at 30 sites along an LHC gradient in the Danube River floodplain. We examined the habitat structure, vegetation cover, and the physical and chemical characteristics of waterbodies. Structural equation models (SEM) were used to explore LHC’s direct and indirect effects, mediated by these environmental factors.

Results showed that LHC did not directly influence *Pelophylax* spp. and *B. bufo* abundance but affected them indirectly and species specifically through environmental drivers. Hydrology negatively and indirectly impacted *Pelophylax* spp. through vegetation cover and the physical and chemical properties of waterbodies. *B. bufo* was affected in a similar way but primarily through habitat structure.

Our findings highlight the importance of understanding species-specific responses to lateral hydrological connectivity in dynamic environments, as river regulation altering LHC can differentially impact amphibian species in riverine floodplains.

**Supplementary Information:**

The online version contains supplementary material available at 10.1007/s10750-026-06110-5.

## Introduction

Riverine floodplain ecosystems have a remarkable diversity and productivity which often surpasses that of purely terrestrial or aquatic ecosystems (Tockner & Stanford, 2002). They are crucial for maintaining high regional biodiversity and play an important role in linking various landscape patches (Amoros & Bornette, 2002; Erős et al., 2019). Floodplains, as dynamic landscapes, are among the most human-impacted ecosystems globally (Habersack et al., 2016). In Central Europe, up to 90% of floodplains are degraded and lack natural water level dynamics due to river regulations and flood protection, with climate change expected to worsen this by causing more extreme floods, droughts, and shorter hydroperiods (Tockner et al., 2008; Hall et al., 2014; Hein et al., 2016).

Riverine floodplains are driven by high-energy flows creating habitat heterogeneity, as well as by the dynamic and variable connectivity with river flows through periodic floodwater inundation (Bunn & Arthington, 2002; Dudgeon & Strayer, 2025). Consequently, aquatic habitats in a riverine floodplain vary in their level of connectivity to the main channel, forming a gradient from water bodies with permanent or frequent connections to those that are isolated or only connected during high or extreme floods (Chaparro et al., 2023). This lateral hydrological connectivity (LHC) plays a key role in shaping habitat connectivity within floodplains, exerting a significant influence on biodiversity patterns and ecosystem processes (Friberg et al., 2017; Hayes et al., 2018; Feng et al., 2024). Hydrology not only directly facilitates the exchange of organisms between rivers and floodplain wetlands but also indirectly impacts aquatic biota by shaping local environmental conditions within wetlands (Funk et al., 2023a, b). These effects include modifications to habitat structure (Bunn and Arthington, 2002; Dudgeon & Strayer, 2025), vegetation (Zhang et al., 2022), and physical and chemical properties of waterbodies (Lizotte et al., 2012) through the transport of water, organic matter, nutrients, and sediment from the river. Consequently, altered hydrology is expected to significantly affect the composition of aquatic communities with different effects on various taxonomic groups, such as macrophytes, fishes, macroinvertebrates, and amphibians. Through both the direct and indirect effects, intermediate levels of LHC are typically linked to the highest biodiversity for aquatic macroinvertebrates in river floodplain systems. Low LHC tends to limit biodiversity for fishes, but can increase it for amphibians by promoting habitat isolation and creating heterogeneous environmental conditions often characterized by lentic, less turbid and more vegetated habitats. On the other hand, high LHC, driven by regular flooding, can reduce biodiversity for amphibians by homogenizing habitats resulting in lotic, turbid, and nutrient-rich environments while its impact on fishes is positive (Tockner et al., 1998; Ward & Tockner, 2001; Thomaz et al., 2007; Bozelli et al., 2015; Fischer et al., 2018; Dube et al., 2019).

Floodplains are crucial for many amphibian species, providing vital terrestrial and aquatic habitats in close proximity to support their complex life cycles (Holgerson et al., 2019; Campos et al., 2020). These species rely on aquatic habitats for breeding, larval development, and metamorphosis, and terrestrial habitats for adult life stages. Consequently, amphibians are particularly sensitive to the loss or alteration of both habitat types and disruptions in the connectivity between them (Becker et al., 2010). Previous studies have shown that, unlike other taxa such as fishes, amphibians are more diverse and abundant in waterbodies that are less connected, more isolated, and heavily vegetated, particularly those that are free of fish (Hamer et al., 2023; Mathwin et al., 2023; Tockner et al., 1998; Ward & Tockner, 2001). Altered LHC, through direct and indirect effects, can severely disrupt population dynamics and compromise the long-term viability of amphibian populations (McGinness et al., 2014). For example, floodplain water bodies with shorter hydroperiods tend to exclude species that require longer development times for metamorphosis, while those with prolonged hydroperiods often exclude species vulnerable to predators like fishes (Baber et al., 2004). Hydroperiod length can strongly influences amphibian life-history traits too, which can profoundly impact populations (Indermaur et al., 2010). In floodplains, a shorter hydroperiod is associated with reduced LHC, while a longer hydroperiod corresponds to increased LHC. Consequently, even small changes in LHC resulting from river regulations and flood protection can significantly affect the hydroperiod, thereby impacting amphibian populations in riverine floodplains (Holgerson et al., 2019; Ocock et al., 2024). For example, the regulation of water flows that support riparian wetlands has diminished breeding opportunities for *Bufo woodhousii* Girard, 1854 and *B. cognatus* Say, 1822 along a river in the USA (Bateman et al., 2008). Although the important role of amphibians in transferring significant biomass and nutrients from wetlands to terrestrial ecosystems is well recognized (Earl et al., 2022), our understanding of amphibian ecology in floodplain wetland systems linked to LHC is still limited compared to other animal groups (Tockner et al., , 1998, 1999, 2006; Ocock et al., 2016). Additionally, studies on amphibian abundance patterns in these wetlands are rare (Ocock et al., 2016; Littlefair et al., 2021; Hamer et al., 2023). This research gap is increasingly urgent, as amphibian populations have been declining worldwide for decades due to various stressors such as emerging diseases, habitat loss, and degradation (Grant et al., 2016; Luedtke et al., 2023). In addition to these factors, a major stressor could be the alteration of river flows, which disrupts natural LHC patterns and, in turn, hydroperiod, potentially impacting amphibian populations. To effectively support wetland amphibians in riverine floodplains, alterations in flood regulation should consider their behaviour, physical traits, and life cycle dependencies on the flood regime.

In expansive waterbodies like riverine floodplains, traditional amphibian sampling methods can be difficult to implement. Methods like larval dipnet surveys, line transect surveys, trap-barrier systems, artificial cover techniques, and visual and auditory encounter surveys can be influenced by a range of biotic and abiotic factors (Schmidt, 2005; Asad et al., 2020). Additionally, a high level of taxonomic expertise is needed to identify eggs, larvae, or even adult individuals (Barata et al., 2017). As a result, the reliability of these traditional methods is often compromised, leading to biased estimates of species occurrence and abundance. In contrast, environmental DNA (eDNA) approaches, which involves the detection of species genetic material directly from environmental samples (soil, sediment, water, air, etc.), are a non-invasive survey technique (Taberlet et al., 2012). It has consistently proved to offer more accurate and representative data on species occurrence and relative abundance, especially in amphibian communities within large rivers and their associated wetland habitats (Svenningsen et al., 2022; Wikston et al., 2023; Sun et al., 2024).

The aim of our study was to investigate the most dominant species of the amphibian community inhabiting the floodplains of the Danube River and explore both the direct and indirect effects of lateral hydrological connectivity. Specifically, we aimed to identify the key factors driving variation in the relative abundance of these amphibians across a gradient of LHC, as well as other environmental drivers—such as local habitat structure, vegetation coverage, and the physical and chemical characteristics of waterbodies—that underpin how LHC affects their relative abundance. To accomplish this, we used eDNA surveys to assess the entire amphibian community and estimate the relative abundance of the target species. Finally, we developed structural equation models (SEMs) for the most abundant species to examine the direct and indirect effects of LHC, with the indirect effects being mediated by environmental factors. To the best of our knowledge, this is the first study to examine the direct effects of LHC and also explore its indirect, underlying determinants on the relative abundance of amphibian species across a complete river-floodplain connectivity gradient using eDNA surveys.

## Materials and methods

### Study area

**Fig. 1:**
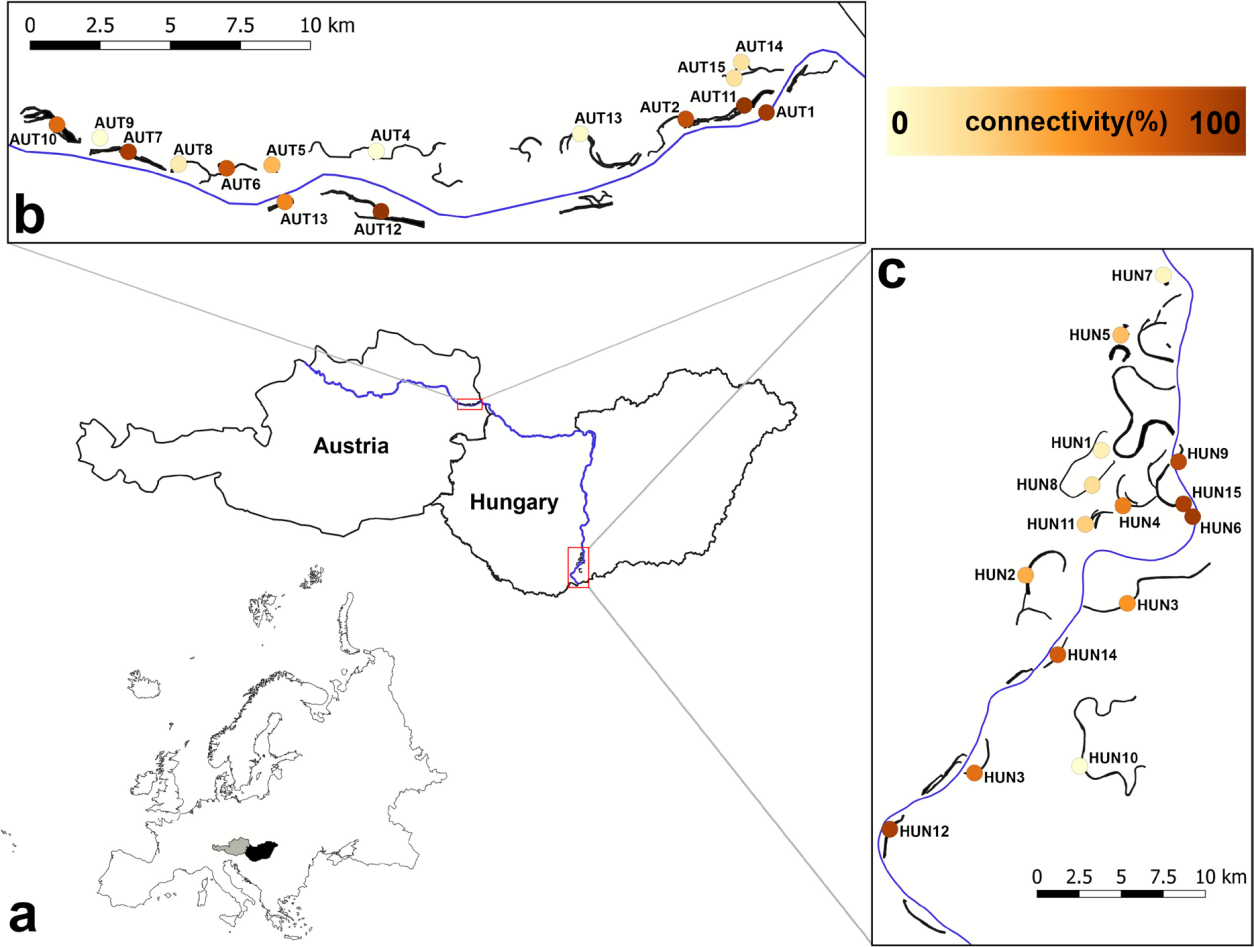
Map of the study area in Europe (a), showing sampling locations in Austria (b) and Hungary (c). Dots are colour-coded according to their connectivity values. The WGS48 coordinates of the sampling locations are provided in Online Resource Table 1

We conducted our study on two floodplain areas along the Danube River in Europe (Fig. 1). Spanning a drainage basin of approximately 800,000 km^2^ with an average discharge of 6,500 m^3^/s at its mouth, the Danube crosses 19 countries, making it the most internationally diverse catchment area in the world (ICPDR, 2024). In the studied segment of the Danube, the river’s mean annual discharge ranges between 1,900 and 2,400 m^3^/s. We studied two floodplain areas, one located in the Donau-Auen National Park in Austria (AUT) and the other in the Danube-Dráva National Park in Hungary (HUN). Both areas host a diverse range of river-floodplain functional habitat types, such as the main channel, side channels connected to the main channel at both ends, and fully isolated oxbow lakes (Erős et al., 2023). Across 11 sampling occasions from 2021 to 2023 (Online Resource Table 1), a total of 15 sites were investigated within the floodplains of AUT and 15 within HUN (Fig. 1). These sites were chosen to represent the entire range of functional habitats, from the main river to the most isolated floodplain ponds.

### eDNA metabarcoding analysis

For eDNA metabarcoding analysis, water samples were collected either from a boat or by carefully wading across various mesohabitats present at each floodplain site. To prevent eDNA cross-contamination between sites, the operator entered the water downstream of the filtration area, holding the sampling device and pointing it directly in the direction of the boat’s movement or the walking path. A VigiDNA 0.45 μm crossflow filtration capsule (SPYGEN) and a peristaltic pump, with disposable sterile tubing attached to a rod, were used to filter the water in situ. The rod, covered with a single-use sterile plastic protective sleeve, was consistently pointed in the direction of movement. An average of 18.31 L of water (ranging from 3 to 94 L) was filtered at each site, depending on the clogging rate of the filtration capsule, with one filter used per site and session. To avoid eDNA degradation, the water in the capsule was drained, and at the end of each filtration, the capsule was refilled with 80 mL of conservation buffer CL1 (SPYGEN).

The eDNA metabarcoding process, involving extraction, amplification using “teleo” and “batra” primers (Valentini et al., 2016) were carried out following the protocol described in Pont et al., (2018). Each field sample underwent twelve PCR replicates to ensure robustness and reliability of the results. Within each PCR replicate, the forward and reverse primer tags remained consistent. Libraries for the high-throughput sequencing were prepared using the TruSeq kit (Illumina) following the manufacturer’s instructions and then they were sequences on a NextSeq 1000 sequencer at Fasteris facilities or at SPYGEN. Extraction and amplification negative controls were performed for each batch of extraction and amplification, and they were sequenced in parallel of the samples (12 amplification per negative control). Subsequently, bioinformatic analysis and taxonomic assignment of sequences were conducted using OBITools package (Boyer et al. 2016), following the methodology outlined in Pont et al., (2023). Initially, the forward and reverse reads were merged, demultiplexed, each samples were then segregated into a distinct dataset, and dereplicated. Sequences shorter than 20 bp, occurring less than 10 times per sample, or identified as “internal” by the obiclean program were excluded. Taxonomic assignments were performed using ecotag program employing a combination of publicly available sequences (Genbank v 247) and a local database (Pont et al., 2023 for fish species and this paper for the amphibian species). Local reference database for amphibians was constructed using the protocol described in (Pont et al., 2023) using “batra” primer instead of “teleo” primers. To address the potential misassignment of sequences to samples caused by tag jumps, all sequences with a frequency of occurrence below 0.001 per sequence per library were excluded. The resulting data were curated for index hopping using a threshold empirically determined for each sequencing batch. This threshold was based on experimental blanks, which included tag combinations absent from the libraries within the batch. To ensure accuracy, six negative extraction controls and one negative PCR control (ultrapure water) were amplified in 12 replicates and sequenced alongside the samples.

Since the most detectable and most abundant species were water frogs (*Pelophylax* spp.) and common toads [*Bufo bufo* (Linnaeus, 1758)] we used their eDNA results for further analyses. Sequences associated with the *Pelophylax* genus could not be definitively identified at the species level (Hofman et al., 2012).

We focused exclusively on the sampling occasions that coincided with the peak breeding season (April–June) of these two species (Tóth, 2015; Bombay, 2024). These data were available only for 2022 and 2023, specifically during sampling occasions T4, T5, T8, and T9 (Online Resource Table 1). This selected dataset was used for subsequent analyses (Online Resource Table 2).

The number of sequence reads for a given amphibian species cannot be directly compared across sites or sampling occasions due to variations in sequencing depth between samples (i.e. the total number of reads across all species). However, the number of positive PCR replicates for a species are comparable, as each site and sampling occasion typically included 12 PCR replicates (Biggs et al., 2015). Pont et al., 2018 demonstrated that the number of positive replicates per species is strongly correlated to the number of reads per species, and thus this metric can be used for relative abundance estimation. In cases where the DNA of the samples was lower and fewer than 12 PCR replicates in total were obtained (e.g. 10 or 11 replicates), the number of positive PCR replicates for each species was proportionally adjusted to a standard total of 12 replicates. This adjustment ensured consistent and comparable results across all sites and occasions. The corrected number of positive PCR replicates was subsequently used as relative abundance in further analyses. For each PCR, the positive detections of three amplicons assigned to *Pelophylax* (*P*. kl. *esculentus* (Linnaeus, 1758) and *P. ridibundus* (Pallas, 1771), *Pelophylax*–Complex 1, and *P. ridibundus* from the Austrian database) were combined to provide a proxy of relative abundance, ranging from 0 to 36 detections per sample (in contrast to 0 to 12 for *B. bufo*). For the final taxa list and the relative abundance of *Pelophylax* spp. and *B. bufo*, see Online Resource Tables 3 and 2.

### Environmental variables

Lateral hydrological connectivity (LHC) was defined as the percentage of the average number of days per year that a waterbody remains connected to the main channel of the Danube (Reckendorfer et al., 2006; Funk et al., 2013; Funk et al., 2023a, b). We calculated LHC using stream gauge data from the Danube covering the period 2000–2016, which encompasses a wide range of hydrological conditions, including high floods, average flows, and low-water periods. Based on these data, we derived stage–discharge relationships and discharge frequency distributions, while site-specific inflow thresholds were determined using a digital terrain model (DTM) processed with the Adaptive Hydraulics (AdH) modelling system (Füstös et al., 2023). Where necessary, the model outputs were updated using field observations from 2021 to 2023. This measure ranges from fully isolated sites (0% connectivity) to fully connected sites (100% connectivity) (Fig. 1 and Online Resource Tables 1 and 4).

The local habitat structure of a waterbody was further characterized by its area, depth, current velocity and by the type of the bank structure. The area of the sampled waterbody (AREA) was measured in km^2^ using the software QGIS. Separate waterbodies were defined based on transversal check dams and natural fords, following the approach of Reckendorfer et al., (2006). The delineation of areal extent was carried out using the boundaries of higher riparian vegetation (woody plants, floodplain forest, and shrubs). Depth (DEPTH) values were determined in metres (m) calculated from 2D hydrodynamic models of the areas of interests (Tritthart, 2005; Tritthart et al., 2019; Füstös et al., 2023; Hawez et al., 2025) in combination with field observations and gauging data from 2021 to 2023. For the Danube main channel, depth information was obtained from regular bed elevation measurements provided by the responsible water management authorities (Austrian River Authority via Donau Österreichische Wasserstraßen GmbH and Hungarian National Water Authority). Current velocity (VELOCITY) was measured in centimetres per second (cm/s) using a water velocity meter. The bank composition was defined by the following variables in the 5 m perimeter of the shoreline: the percentage of trees or large bushes (WOODY), the percentage of herbaceous vegetation (HERBACEOUS), and the percentage of artificial bank structures such as rip-rap or concrete (ARTIFICIAL).

Vegetation within the waterbodies was characterized by visually estimating the percentage composition of the following variables for the whole waterbody: % emergent (EMVEG), % submerged (SUBVEG), % floating vegetation (FLOATVEG), % floating algae (ALGAE), and % open water habitat (OPENWATER).

The physical and chemical variables of each waterbody were measured in two ways. Water temperature (WTEMP), pH (PH), conductivity (COND), and dissolved oxygen (OXY) were estimated using portable sensors in the field, while water samples were collected for laboratory analysis to determine chlorophyll-a (CHLO-A), total dissolved phosphorus (TP), chromophoric dissolved organic matter (CDOM), and total suspended solids (TSS). Chlorophyll-a was quantified by filtering 300–2000 mL of water through pre-combusted GF/C filters (Whatman). Afterwards, chlorophyll-a was extracted using cold 90% acetone, and its absorption was measured with a spectrophotometer (Lorenzen, 1967; Jeffrey & Humphrey, 1975). Total phosphorus was digested with persulfate and analysed following the method outlined by Eaton and Franson (2005), in accordance with ÖNORM EN ISO 15681–2. Following Cuthbert and Del Giorgio (1992), water was filtered through 0.45 μm Teflon membrane filters, and light absorbance at 440 nm was measured to estimate CDOM. To quantify total suspended solids, 300–2000 mL of sample water was filtered through pre-combusted GF/F filters (Whatman). The filters were then dried at 80 °C for 24 h, and the TSS was determined by weighing the dried filters.

Fish presence/absence was not included as a variable in the analyses because eDNA data from a parallel study at the same sites indicated that fish were present in all sampled waterbodies, making this factor homogeneous across sites. Consequently, including it would not contribute explanatory power to the models.

The averages of the environmental variables (except AREA) from sampling occasions T4, T5, T8, and T9 (Online Resource Table 2), were used for data analysis. They are summarized in Online Resource Table 4.

### Data analyses

To simplify the dataset and reduce the number of variables to a smaller set of independent explanatory variables, representing environmental gradients (Peres-Neto et al., 2003; Czeglédi et al., 2020), we conducted principal component analyses (PCA) separately on three predictor groups: local habitat structure (STRUCT = AREA, DEPTH, VELOCITY, WOODY, HERBACEOUS, ARTIFICIAL), vegetation (VEG = EMVEG, SUBVEG, FLOATVEG, ALGAE, OPENWATER), and physical and chemical (PHYSCHEM = WTEMP, PH, OXY, COND, TSS, TP, CDOM, CHLO-A) predictors. Prior to the PCA, all variables were standardized using the *scale()* function in R, so that each had a mean of 0 and a standard deviation of 1, ensuring equal contribution to the analysis. Since the first axis in the PCAs described the main environmental gradients (see Results for details), further PCA axes were not used in this study for model parsimony (Online Resource Tables 5–7).

Space can be considered as a factor shaping ecological structures. To account for spatial autocorrelation effects we used principal coordinates of neighbour matrices (PCNM) to create spatial descriptors, which allowed for the quantification of spatial relationships among sites (Dray et al., 2006). To achieve this, we calculated the direct shortest Euclidean distance between the sampling sites using their geocoordinates. PCNM variables having positive eigenvalues were retained for further models (Borcard & Legendre, 2002; Borcard et al., 2004; Dray et al., 2006). Linear regression models, employing forward selection, identified three PCNM variables (PCNM1, PCNM4, and PCNM5) as significant due to their association with the PHYSCHEM PC and the relative abundance of *B. bufo* (Online Resource Table 8). All these three PCNM variables within the spatial predictor group were retained for subsequent analyses and incorporated into the models for both species.

We constructed structural equation models (SEMs) to assess the impact of LHC on the relative abundance of *Pelophylax* spp. and *B. bufo*, accounting for both direct and indirect effects through environmental drivers like STRUCT, VEG, and PHYSCHEM. PCNM1, PCNM4, and PCNM5 were also incorporated into the SEMs, which represented direct effects on LHC, VEG, STRUCT, PHYSCHEM and the relative abundance of *B. bufo* in order to account for potential spatial autocorrelations. We conducted all analyses separately for the two anurans with the same SEM designs, except that we incorporated PCNM5 as a direct effect on *B. bufo*. In creating the models, we assessed the data, analysis objectives, sample size, and model complexity, opting to simplify them to eight SEM parameters and exclude latent variables due to the small sample size (Grace et al., 2012).

The fit of the SEMs was assessed using Chi-square tests, the Comparative Fit Index (CFI), the Tucker–Lewis Index (TLI), the Root Mean Square Error of Approximation (RMSEA), and the Standardized Root Mean Square Residual (SRMR). A model was deemed to have a good fit if the Chi-square P value exceeded 0.05. Higher CFI and TLI values indicate better fit (CFI/TLI < 0.90 = poor fit; CFI/TLI ≥ 0.90 = acceptable fit; CFI/TLI ≥ 0.95 = good fit). RMSEA values range from 0 to 1, with lower values reflecting better fit (RMSEA > 0.10 = very poor fit; RMSEA > 0.08 = poor fit; 0.05 < RMSEA ≤ 0.08 = acceptable fit; RMSEA ≤ 0.05 = good fit). Similarly, SRMR values range from 0 to 1, with lower values denoting better fit (SRMR > 0.08 = poor fit; SRMR ≤ 0.08 = good fit; SRMR ≤ 0.05 = excellent fit).

Data analyses were performed using R (R Core Team, 2021). The “pcnm” function from “vegan” package was utilized for PCNM analysis (Dray et al., 2006), the “stats” package for PCA analyses (R Core Team, 2021), and the “lavaan” package for constructing SEMs (Rosseel, 2012).

## Results

Using eDNA metabarcoding, a total of 10 amphibian species were identified across 30 sampling points over 11 sampling occasions between 2021 and 2023 (Online Resource Table 3). The most frequently detected species were the *Pelophylax* spp., found at 96.67% of the sites, and *B. bufo*, recorded at 83.3% of the sites (Online Resource Table 2).

The first principal component axes derived from the principal component analyses of local habitat structure (STRUCT), vegetation (VEG), and physical and chemical features (PHYSCHEM) each represented distinct environmental gradients. For STRUCT, PC1 accounted for 54% of the total variance, capturing a gradient from large, deep, fast-flowing, artificial banked waterbodies to small, shallow, slow-moving, and more natural habitats. This gradient was defined by high negative loadings for area (-0.51), depth (-0.44), velocity (-0.39), and artificial structures (-0.51), contrasted with a positive loading for herbaceous bank composition (0.30) (Online Resource Table 5). The PC1 of VEG explained 39% of the variance and described a gradient in vegetation cover and composition. High positive loadings for emergent vegetation (0.52), submerged vegetation (0.38), and surface algae (0.33), contrasted with a strong negative loading for open water (-0.69), indicating a transition from densely vegetated habitats dominated by plants and algae to sparsely vegetated open water environments (Online Resource Table 6). For PHYSCHEM, PC1 accounted for 42% of the total variance and reflected a gradient in nutrient and organic matter concentrations. High positive loadings on total phosphorus (0.50), CDOM (0.49), and chlorophyll-a (0.41), contrasted with negative loadings on pH (-0.39) and dissolved oxygen (-0.33). This gradient indicated a shift from nutrient- and organic matter-rich (eutrophic) environments to nutrient-poor, oxygen-rich (oligotrophic) habitats with low pH (Online Resource Table 7).

The SEM fit was considered good and acceptable for the relative abundance of the *Pelophylax* spp. (Chi-square p = 0.545, CFI = 1.000, TLI = 1.035; RMSEA = 0.000; SRMR = 0.066), as well as for the relative abundance of *B. bufo* (Chi-square p = 0.270, CFI = 0.963, TLI = 0.925; RMSEA = 0.086; SRMR = 0.066). The SEMs explained 39.1% and 32.4% of the variation in relative abundance of *Pelophylax* spp. and *B. bufo,* respectively.

**Fig. 2:**
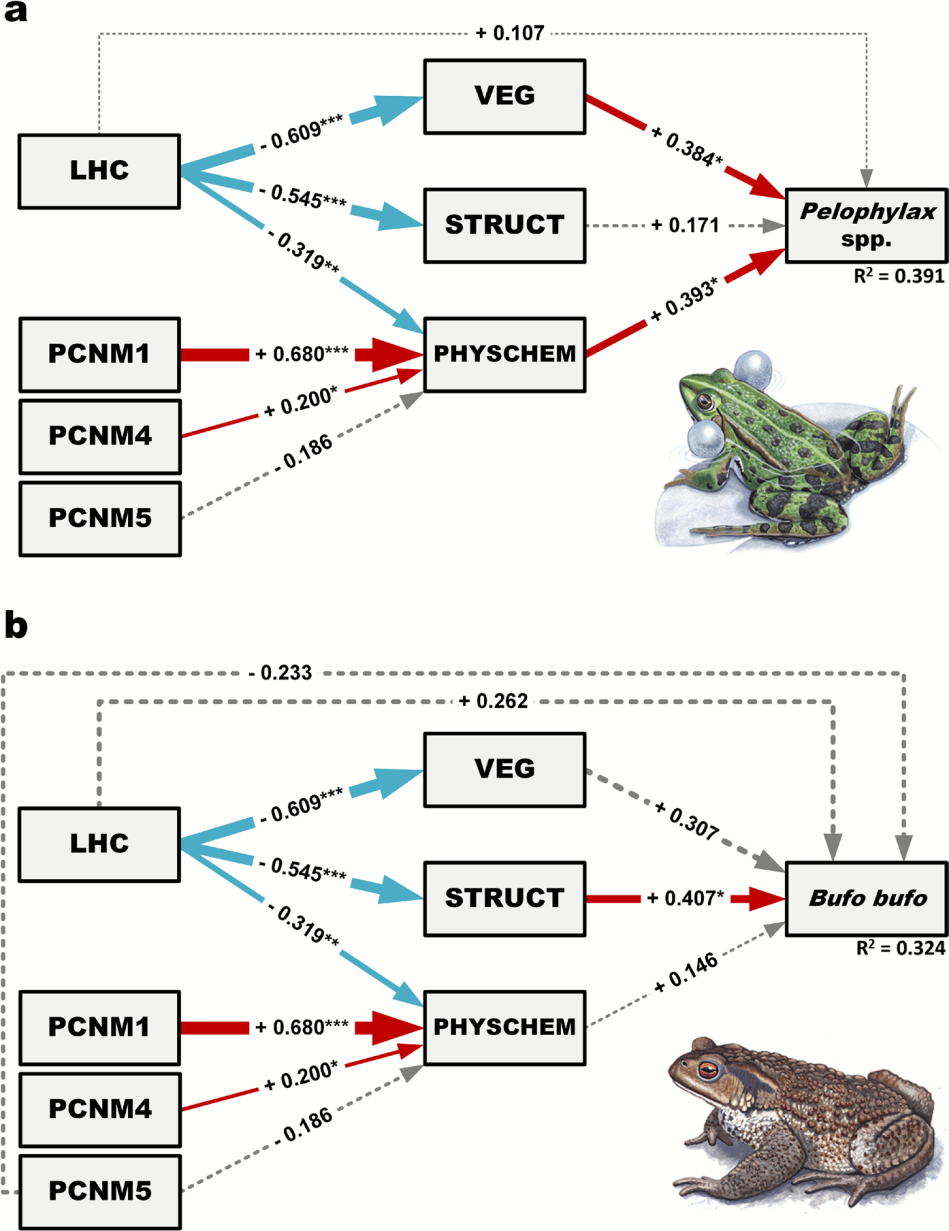
Structural equation models illustrating the direct and indirect effects of lateral hydrological connectivity (LHC), vegetation cover (VEG), local habitat structure (STRUCT), water physical and chemical characteristics and spatial variables (PCNM1, PCNM4, PCNM5) on the relative abundance of the *Pelophylax* spp. (a) and *Bufo bufo* (a). Solid blue lines denote significant negative relationships, solid red lines indicate positive ones, and grey dashed lines represent non-significant connections. The thickness of the lines corresponds to the strength of the regression (standardized regression coefficients). Asterisks denote significance levels: * P < 0.05; ** P < 0.01; *** P < 0.001. Illustrations of *Pelophylax* spp. and *B. bufo*, provided by Márton Zsoldos

The direct effects of LHC on the environmental drivers (VEG, STRUCT, PHYSCHEM) were statistically significant, though their magnitude and direction varied. Specifically, LHC had a negative direct effect on PHYSCHEM (β = -0.319, p = 0.002), indicating that waterbodies with greater connectivity to the main channel of the Danube contain less phosphorus, organic matter, and algae. This reflects a shift towards more oligotrophic conditions in highly connected waterbodies. Similarly, LHC showed a significant negative direct effect on VEG (β = -0.609, p < 0.001), suggesting that increased connectivity to the main channel of the Danube leads to a reduction in vegetation within waterbodies, resulting in more open water conditions. Furthermore, LHC also exerted a significant negative direct effect on STRUCT (β = -0.545, p < 0.001), indicating that increased connectivity to the main channel of the Danube is associated with larger, deeper, faster-flowing waterbodies with more artificial banks. In summary, LHC exhibited a strong negative impact on all environmental driver variables. As the same SEMs were applied to both species, the findings on the direct effects of LHC on environmental drivers are consistent across both models (Fig. 2 and Online Resource Tables 9 and 11).

For the relative abundance of *Pelophylax* spp., the direct effect of LHC was not significant (β = 0.107, p = 0.623), indicating no direct influence on the relative abundance of the species complex (Fig. 2a and Online Resource Table 9). However, LHC exerted a significant negative overall indirect effect (β = -0.453, p = 0.008), primarily mediated through VEG, STRUCT, and PHYSCHEM. This suggests that LHC impacts *Pelophylax* spp. relative abundance indirectly via changes in vegetation, local habitat structure, and water physiochemical features. Consequently, the total effect of LHC on *Pelophylax* spp. relative abundance was also negative and significant (β = -0.346, p = 0.035), highlighting that its cumulative impact is driven predominantly by these indirect pathways (Online Resource Table 10). In addition, we observed marginally significant negative indirect effects of LHC through PHYSCHEM (β = -0.125, p = 0.056) and VEG (β = -0.234, p = 0.061), suggesting a tendency for LHC to negatively impact the relative abundance of *Pelophylax* spp. by altering the vegetation and the physical and chemical characteristics of the waterbody (Online Resource Table 10).

PHYSCHEM exerted a significant positive direct effect on the *Pelophylax* spp. relative abundance (β = 0.393, p = 0.014), suggesting that waterbodies with higher levels of nutrients, organic matter, and algae are associated with higher abundances of these frogs. Similarly, VEG had a significant positive direct effect (β = 0.384, p = 0.036), indicating that waterbodies with more emergent, submerged vegetation and surface algae tend to support more abundant *Pelophylax* spp. greater toad abundances. In contrast, STRUCT did not have a significant direct effect on the relative abundance of *Pelophylax* frogs (β = 0.175, p = 0.334) (Fig. 2a and Online Resource Table 9). These findings suggest that LHC negatively affects *Pelophylax* spp. through its influence on environmental factors, particularly through PHYSCHEM and VEG, rather than via a direct relationship.

The direct effect of LHC on *B. bufo* relative abundance was not significant (β = 0.262, p = 0.253), indicating that there is no direct influence (Fig. 2b and Online Resource Table 11). However, the overall indirect effect of LHC on *B. bufo* was negative and statistically significant (β = -0.455, p = 0.012), suggesting that LHC negatively influences the relative abundance of the species primarily through environmental drivers. The indirect effect through STRUCT was marginally significant (β = -0.222, p = 0.060), indicating that LHC negatively impacts *B. bufo* relative abundance by modifying the local habitat structure (Online Resource Table 12).

For the relative abundance of *B. bufo*, STRUCT (β = 0.407, p = 0.026) had a significant positive direct effect, suggesting that smaller, shallower, slower-moving waterbodies with a higher proportion of herbaceous bank vegetation are associated with more abundant common toad populations. In contrast to *Pelophylax* spp. SEMs, PHYSCHEM (β = 0.146, p = 0.395), and VEG (β = 0.307, p = 0.111) did not show a significant direct effect on *B. bufo* relative abundance (Fig. 2b and Online Resource Table 11). These findings suggest that LHC negatively affects the relative abundance of *B. bufo* through its influence on environmental factors, particularly through local habitat structure.

In both SEMs, PCNM1 (β = 0.680, p < 0.001), and PCNM4 (β = 0.200, p = 0.048) had significant positive direct effects on PHYSCHEM. However, PCNM5 did not have a significant direct effect on either PHYSCHEM or the relative abundance of *B. bufo* (see Fig. 2 and Online Resource Tables 9 and 11).

## Discussion

Riverine floodplains are composed of various aquatic habitats, including fast-flowing mainstem rivers, slow-moving side channels, and calm oxbows, ponds, and seasonally flooded fields (Holgerson et al., 2019). Numerous studies have shown that fish species are more diverse and abundant in more connected and permanent waterbodies (Erős et al., 2024; Feng et al., 2024), whereas amphibian species richness and abundance tend to peak in more isolated and temporary waterbodies (Tockner et al., 1998; Ocock et al., 2016; Littlefair et al., 2021). For instance, the abundance of amphibians decreases with lateral hydrological connectivity in the floodplains of both the Hungarian (Hamer et al., 2023) and the Austrian Danube (Tockner et al., 1998). Supporting our predictions and previous studies on amphibians, we also observed a negative correlation between LHC and the relative abundance of two anuran species. However, this relationship was indirect, indicating that the specie’s relative abundance is primarily driven by local environmental factors shaping habitat heterogeneity (Thomaz et al., 2007; Bozelli et al., 2015; Fischer et al., 2018; Dube et al., 2019), rather than by hydrological connectivity directly influencing their dispersal opportunities. Amphibian species presence is often driven by local environmental factors, including pond characteristics such as hydroperiod, aquatic vegetation coverage, and predator presence (Semlitsch, 2000). Multiple studies have shown a positive correlation between habitat heterogeneity and amphibian richness and abundance (Hartel et al., 2007; Vasconcelos et al., 2009; Jeliazkov et al., 2014; Dick et al., 2017; Li et al., 2018), emphasizing the role of diverse, well-structured habitats in supporting amphibian populations. In our study, local environmental factors—including habitat structure, aquatic vegetation coverage, and water physical and chemical properties—had direct yet distinct effects on the relative abundance of *Pelophylax* spp. and *B. bufo*, indicating species-specific responses to these habitat conditions.

Firstly, LHC exhibited a negative influence on local habitat structure, suggesting that increased connectivity to the main channel is associated with larger, deeper, and lotic waterbodies, whereas more isolated waterbodies tend to be smaller, shallower, and lentic. Our findings indicate that the relative abundance of *Pelophylax* spp. is not significantly influenced by these habitat characteristics, implying that their breeding behaviour is not strictly dependent on small, shallow, lentic waters, as also reported in previous studies (Korzikov & Aleksanov, 2018; Crnobrnja-Isailović et al., 2022). In contrast, local habitat structure played a crucial role in determining the relative abundance of *B. bufo*, with higher abundances observed in smaller, shallower, and more lentic waterbodies. As an explosive breeder, *B. buf*o relies on synchronized arrival at breeding ponds to maximize reproductive success and enhance the survival of tadpoles through predator dilution or selfish herd geometry (Grant et al., 2013; Jarvis et al., 2021). Weather is a key trigger for migration to breeding ponds (Reading, 2010; Dalpasso et al., 2023), and since smaller, shallower, and non-flowing waterbodies warm earlier in the spring (Lizotte et al., 2012; Chaparro et al. 2023) compared to larger lentic waters, they may create favourable conditions for an explosive breeder like *B. bufo* (Jarvis et al., 2021). Additionally, spiny common toads (*B. spinosus* Daudin, 1803) reach their largest body size at metamorphosis in ponds with short hydroperiods (low LHC), which are warmer, more isolated, and characterized by lower intraspecific competition and reduced predation risk (Indermaur et al., 2010). This is consistent with our observations and previous studies, which indicate that common toads tend to occur more frequently in relatively isolated ponds.

In addition to its influence on local habitat structure, LHC had a negative impact on vegetation coverage, suggesting that increased connectivity to the main channel reduces vegetation, resulting in more open water conditions, whereas more isolated waterbodies support greater vegetation heterogeneity with more emergent and submerged vegetation. Anuran species display distinct habitat preferences, largely shaped by vegetation characteristics (Burrow & Maerz, 2022). In wetland ecosystems, the presence, absence, density, diversity, and structural complexity of aquatic vegetation play a crucial role in determining amphibian distribution, abundance, species richness, and community composition (Hazell et al., 2001; Wassens et al., 2010; McGinness et al., 2014). For example, a study of 133 ponds in north-central Hungary found a positive correlation between amphibian richness and the abundance of aquatic vegetation (Vági et al., 2013). Densely vegetated waters create microhabitats that offer essential resources for anuran larvae by promoting algal growth, a key food source for tadpoles, and for adults by providing habitat for a diverse range of macroinvertebrate prey (Burrow and Maerz, 2022). Additionally, they create optimal reproductive conditions by providing structural support for egg deposition (Hartel, 2004; Hartel et al., 2007). Furthermore, pond vegetation plays a crucial role in fostering habitat diversity and serves as an important refuge against predation (Teplitsky et al., 2003). Our results showed that vegetation cover plays a crucial role in influencing *Pelophylax* spp. relative abundance, with waterbodies containing more emergent and submerged vegetation, along with algae, supporting greater water frog abundances. This highlights the importance of vegetation in offering both shelter and food resources for this species. In contrast, we observed that the relative abundance of *B. bufo* was unaffected by vegetation cover. Previous studies have shown that *B. bufo* is not sensitive to the presence of fish, likely due to unique larval adaptations. These tadpoles often coexist with predatory fish either because their toxicity makes them unpalatable (Üveges et al., 2021), they form large aggregations as a defence mechanism (Kloskowski, 2009), or they exhibit explosive breeding behaviour, spending little time in aquatic habitats (Jarvis et al., 2021). We are aware that fish predation can significantly influence amphibian communities. eDNA data from a parallel study at the same sites indicated that fish were present in all sampled waterbodies, making this factor uniform across sites. Consequently, fish presence was not included as a variable in our models. Nonetheless, we assume that dense vegetation may provide *Pelophylax* spp. with shelter from fish predation, contributing to their higher abundances in vegetated waters, whereas *B. bufo* likely remains unaffected due to larval adaptations such as toxicity, aggregation, and explosive breeding behaviour. We acknowledge that the absence of species- or density-specific fish data represents a limitation of our study and should be considered when interpreting the results.

Finally, LHC showed a negative correlation with the physical and chemical properties of waterbodies. Higher connectivity was associated with lower levels of phosphorus, organic matter, and algae, along with increased oxygen and lower pH (Hein et al., 2004; Funk et al, 2023a, b). This suggests that isolated waterbodies are more susceptible to eutrophication. This finding aligns with Lizotte et al., (2012), who reported that artificial flooding helps stabilize water quality by regulating temperature, oxygen levels, pH, and nutrient concentrations. Changes in the physical and chemical properties of the waterbody are expected to significantly shape aquatic community composition, with particularly strong effects on sensitive groups like amphibians due to their ectothermic nature and complex life cycles (Campos et al., 2020; Larsen, 2021; Bofill & Blom, 2024). Consistent with this, our SEM models revealed that waterbody physical and chemical characteristics positively influenced *Pelophylax* spp. relative abundance, suggesting that increased nutrients, organic matter, and algae support greater abundance of water frogs. Both water frogs and common toads rely on nutrient-rich, algae-abundant water bodies to support their metamorphosis. However, our analysis found that these factors did not significantly influence the relative abundance of *B. bufo*, likely due to differences in the breeding strategies of the two species. Common toads employ an explosive breeding strategy (Jarvis et al., 2021), with tadpoles that undergo rapid metamorphosis. In contrast, water frogs have a prolonged larval period, remaining in the water until summer before completing their transformation (Beebee & Griffiths, 2000; Tóth, 2015; Bombay, 2024). This extended time in the aquatic environment requires sustained access to algae-rich habitats, as their tadpoles depend on abundant algae for growth. Isolated, nutrient-rich water bodies with higher organic matter levels foster increased algal growth and detritus accumulation, providing essential food resources for water frog tadpoles (Breka et al., 2024; Flecker et al. 1999; Kupferberg, 1997).

Our PCNM analysis revealed that neither the relative abundance of *Pelophylax* spp. nor *B. bufo* displayed a direct spatial structure. However, we identified indirect spatial effects on *Pelophylax* spp. through the physical and chemical properties of the waterbodies. Both larger-scale processes and finer-scale processes played a crucial role in shaping these characteristics. The significant direct influence of regional-scale processes (PCNM1) on the physical and chemical properties of the waterbodies can be attributed to the geographic separation between the Austrian and Hungarian sampling sites. The Austrian sites, located upstream along the Danube River, contrast with the Hungarian sites downstream, leading to distinct differences in water properties. Additionally, we found that a smaller-order spatial variable (PCNM4) also influenced these water properties, indicating the role of finer-scale spatial processes. This underscores the importance of considering both broad and localized spatial influences when managing river-floodplain systems.

## Conclusion

In summary, we showed that lateral hydrological connectivity influences the relative abundance of two anuran species living in the floodplains of the Danube River in Austria and Hungary, using eDNA surveys. Our study identified the main factors driving variations in the relative abundance of *Pelophylax* frogs and *B. bufo* along a gradient of LHC, as well as the mediating factors underlying these effects. Our SEM models revealed that LHC did not directly affect the relative abundance of *Pelophylax* spp. and *B. bufo*. Instead, it influences their abundance through indirect pathways, which were mediated by environmental variables such as local habitat structure, vegetation, and the physical and chemical properties of the waterbody. Our findings revealed that these pathways differ between the two species: the relative abundance of common toads are primarily determined by the local habitat structure and vegetation, while water frogs show a stronger response to the nutrient and algal richness of the waterbody. This highlights species-specific responses to lateral hydrological connectivity and offers new insights into how hydrology can influence amphibian populations. Amphibians play a vital role in riverine floodplain wetlands, acting as key links in the food web by facilitating energy transfer between aquatic and terrestrial ecosystems. They serve as predators of invertebrates while also being prey for fish, reptiles, other amphibians, mammals, and birds. Given that river regulation poses a significant threat to amphibian communities and individual species (Mathwin et al., 2023), a comprehensive approach is essential to understand species-specific responses to a range of environmental factors in these dynamic ecosystems. Beyond hydrological connectivity, variables such as habitat structure, vegetation cover and nutrient availability also play crucial roles in shaping amphibian populations (Ocock et al., 2024). Integrating these factors provides a clearer understanding of how river regulation impacts amphibian populations.

## Supplementary Information

Below is the link to the electronic supplementary material.

Supplementary file1 (PDF 666 KB)

## Data Availability

All data supporting the findings of this study and sequences for amphibian reference databases and all Illumina raw sequences data will be available after acceptance in a data repository.
