# Supplementary material for: Effects of lateral hydrological connectivity on the relative abundance of water frogs (Pelophylax spp.) and common toads (Bufo bufo) using eDNA surveys
Source: Hydrobiologia. 2026 Jan 29;853(9):2751–67. doi: 10.1007/s10750-026-06110-5 (PMC13098841; doi:10.1007/s10750-026-06110-5)
Supplement: Supplementary file 1 — Supplementary file1 (PDF 666 KB) [file 10750_2026_6110_MOESM1_ESM.pdf]

Supplementary material for "Effects of lateral hydrological connectivity on the relative abundance of water frogs (*Pelophylax* spp.) and common toads (*Bufo bufo*) using eDNA surveys"

Journal: Hydrobiology

Authors and affiliations:
Boglárka Mészáros (ORCID: 0000-0003-0005-8678) [1, 2 *]
Andrea Funk (ORCID: 0000-0002-0568-1234) [3]
Thomas Hein (ORCID: 0000-0002-7767-4607) [3]
Lukas Landler (ORCID: 0000-0002-5638-7924) [4]
Paul Meulenbroek (ORCID: 0000-0001-9232-2792) [3]
Didier Pont (ORCID: 0000-0001-5187-0135) [5]
Alice Valentini (ORCID: 0000-0001-5829-5479) [6]
Dénes Schmera (ORCID: 0000-0003-1248-8413) [1, 2]
István Czeglédi (ORCID: 0000-0002-0244-7987) [1, 2 †]
Tibor Erős (ORCID: 0000-0002-2252-3115) [1, 2 †]

[1] HUN-REN Balaton Limnological Research Institute, Klebelsberg Kuno street 3., H-8237 Tihany, Hungary
[2] National Laboratory for Water Science and Water Security, HUN-REN Balaton Limnological Research Institute, Tihany, Hungary
[3] Christian Doppler Laboratory for Meta Ecosystem Dynamics in Riverine Landscapes, BOKU University, Vienna, Austria
[4] Institute of Zoology, BOKU University, Vienna, Austria
[5] Institute of Hydrobiology and Aquatic Ecosystem Management, BOKU University, Vienna, Austria
[6] SPYGEN, Savoie Technolac, Le Bourget du Lac, France

† Contributed equally
*Corresponding author:
e-mail: meszaros.boglarka@blki.hun-ren.hu

Table 1 The WGS coordinates of the sampling sites from Austria (AUT1-11) and Hungary (HUN1-11) with the lateral hydrological connectivity (%) values and date of sampling occasions (T1-T11).

Site	Latitude	Longitude	Connectivity	T1	T2	T3	T4	T5	T6	T7	T8	T9	T10	T11
AUT1	48.14227575	16.74546428	100.00	12/08/2021	21/09/2021	14/03/2022	09/05/2022	23/06/2022	22/08/2022	21/10/2022	03/04/2023	26/06/2023	28/08/2023	28/09/2023
AUT2	48.14943163	16.90419604	66.85	05/08/2021	15/09/2021	08/03/2022	06/05/2022	24/06/2022	24/08/2022	18/10/2022	04/04/2023	28/06/2023	28/08/2023	25/09/2023
AUT3	48.14275672	16.8542813	0.55	06/08/2021	10/09/2021	08/03/2022	NA	21/06/2022	23/08/2022	19/10/2022	03/04/2023	30/06/2023	29/08/2023	25/09/2023
AUT4	48.13735576	16.75616986	0.00	05/08/2021	09/09/2021	07/03/2022	06/05/2022	21/06/2022	24/08/2022	19/10/2022	03/04/2023	26/06/2023	29/08/2023	25/09/2023
AUT5	48.13144907	16.70679128	13.15	09/08/2021	14/09/2021	14/03/2022	NA	NA	NA	NA	NA	NA	NA	27/09/2023
AUT6	48.12972367	16.68614794	58.90	NA	14/09/2021	14/03/2022	09/05/2022	23/06/2022	22/08/2022	21/10/2022	06/04/2023	26/06/2023	28/08/2023	27/09/2023
AUT7	48.13070745	16.66247579	2.19	09/08/2021	14/09/2021	14/03/2022	09/05/2022	23/06/2022	22/08/2022	21/10/2022	06/04/2023	26/06/2023	28/08/2023	27/09/2023
AUT8	48.13339202	16.63808902	100.00	NA	15/09/2021	07/03/2022	06/05/2022	23/06/2022	22/08/2022	19/10/2022	06/04/2023	28/06/2023	28/08/2023	27/09/2023
AUT9	48.13857479	16.62416198	10.01	11/08/2021	09/09/2021	NA	NA	NA	NA	NA	NA	NA	NA	NA
AUT10	48.14273357	16.60155092	47.67	11/08/2021	15/09/2021	07/03/2022	06/05/2022	24/06/2022	22/08/2022	19/10/2022	06/04/2023	30/06/2023	28/08/2023	27/09/2023
AUT11	48.15489902	16.93255462	93.97	NA	21/09/2021	08/03/2022	04/05/2022	21/06/2022	23/08/2022	18/10/2022	03/04/2023	28/06/2023	28/08/2023	25/09/2023
AUT12	48.11765036	16.76074339	47.40	NA	16/09/2021	07/03/2022	04/05/2022	21/06/2022	23/08/2022	18/10/2022	04/04/2023	30/06/2023	28/08/2023	25/09/2023
AUT13	48.1191954	16.71223434	24.38	10/08/2021	16/09/2021	07/03/2022	04/05/2022	21/06/2022	23/08/2022	18/10/2022	04/04/2023	30/06/2023	28/08/2023	25/09/2023
AUT14	48.16672235	16.9302803	3.29	06/08/2021	10/09/2021	08/03/2022	04/05/2022	21/06/2022	23/08/2022	18/10/2022	03/04/2023	28/06/2023	29/08/2023	25/09/2023
AUT15	48.1657641	16.93742345	3.29	06/08/2021	10/09/2021	08/03/2022	NA	24/06/2022	24/08/2022	18/10/2022	04/04/2023	26/06/2023	28/08/2023	25/09/2023
HUN1	46.22717231	18.85533987	2.00	10/08/2021	24/09/2021	09/03/2022	03/05/2022	23/06/2022	18/08/2022	19/10/2022	29/03/2023	13/06/2023	29/08/2023	19/09/2023
HUN2	46.1602602	18.79575556	15.00	19/07/2021	24/09/2021	09/03/2022	03/05/2022	23/06/2022	18/08/2022	19/10/2022	NA	13/06/2023	29/08/2023	19/09/2023
HUN3	46.05373664	18.7566235	45.00	11/08/2021	23/09/2021	10/03/2022	04/05/2022	24/06/2022	19/08/2022	20/10/2022	30/03/2023	14/06/2023	30/08/2023	20/09/2023
HUN4	46.19670566	18.87319593	25.00	10/08/2021	24/09/2021	09/03/2022	03/05/2022	23/06/2022	NA	19/10/2022	29/03/2023	13/06/2023	29/08/2023	19/09/2023
HUN5	46.28924902	18.8693408	10.00	09/08/2021	22/09/2021	09/03/2022	03/05/2022	23/06/2022	18/08/2022	19/10/2022	29/03/2023	13/06/2023	29/08/2023	19/09/2023
HUN6	46.1917723	18.92454561	100.00	19/07/2021	22/09/2021	09/03/2022	03/05/2022	23/06/2022	18/08/2022	19/10/2022	29/03/2023	13/06/2023	29/08/2023	19/09/2023
HUN7	46.32051185	18.90189222	1.50	09/08/2021	22/09/2021	09/03/2022	03/05/2022	23/06/2022	NA	19/10/2022	29/03/2023	13/06/2023	NA	NA
HUN8	46.207495	18.84824471	5.00	12/08/2021	24/09/2021	NA	NA	NA	NA	NA	NA	13/06/2023	NA	NA
HUN9	46.22150797	18.91285219	70.00	11/08/2021	22/09/2021	10/03/2022	03/05/2022	23/06/2022	18/08/2022	20/10/2022	30/03/2023	14/06/2023	30/08/2023	20/09/2023
HUN10	46.05865226	18.83893356	0.00	19/07/2021	23/09/2021	10/03/2022	04/05/2022	24/06/2022	19/08/2022	20/10/2022	30/03/2023	14/06/2023	30/08/2023	20/09/2023
HUN11	46.18706503	18.84300972	8.00	19/07/2021	24/09/2021	NA	NA	NA	NA	NA	NA	13/06/2023	29/08/2023	19/09/2023
HUN12	46.02367491	18.69189516	90.00	19/07/2021	23/09/2021	10/03/2022	04/05/2022	24/06/2022	19/08/2022	20/10/2022	30/03/2023	14/06/2023	30/08/2023	20/09/2023
HUN13	46.14503032	18.87674064	18.00	11/08/2021	23/09/2021	10/03/2022	04/05/2022	24/06/2022	19/08/2022	20/10/2022	30/03/2023	14/06/2023	30/08/2023	20/09/2023
HUN14	46.11720481	18.82022287	50.00	11/08/2021	23/09/2021	10/03/2022	04/05/2022	24/06/2022	NA	20/10/2022	30/03/2023	14/06/2023	30/08/2023	20/09/2023
HUN15	46.19729016	18.91959731	90.00	11/08/2021	22/09/2021	09/03/2022	03/05/2022	23/06/2022	18/08/2022	19/10/2022	29/03/2023	13/06/2023	29/08/2023	19/09/2023

Table 2 The relative abundance of the *Pelophylax* spp. and *B. bufo* for each site and sampling occasion was determined based on the number of positive PCR replicates. The highlighted cells represent the sampling occasions that coincide with the breeding season of both species. For a given PCR, the detections of three amplicons assigned to *Pelophylax* kl. *esculentus* and *P. ridibundus*, *Pelophylax* - Complex 1 and *P. ridibundus* (Austrian database) were cumulated, giving a proxy of relative abundance ranging from 0 to 36 positive detections per sample (instead of 0 to 12 for *B. bufo*).

	Bufo bufo											***Pelophylax* kl. *esclentus* and *P. ridibundus complex***										
**Site**	**T1**	**T2**	**T3**	**T4**	**T5**	**T6**	**T7**	**T8**	**T9**	**T10**	**T11**	**T1**	**T2**	**T3**	**T4**	**T5**	**T6**	**T7**	**T8**	**T9**	**T10**	**T11**
AUT1	0	0	0	0	0	1	1	0	0	0	1	0	0	0	0	0	0	0	0	0	0	0
AUT2	0	0	0	3	0	0	0	5	0	0	0	0	0	0	0	4	3	2	2	4	2	0
AUT3	0	0	0	0	0	0	0	10	0	0	0	0	0	0	0	1	0	0	0	1	0	0
AUT4	0	0	0	12	2	1	0	12	0	0	0	2	14	10	1	17	12	0	9	21	21	12
AUT5	0	0	0	na	na	na	na	9	na	na	0	21	5	12	na	na	na	na	7	na	na	2
AUT6	0	0	0	5	6	7	0	12	11	0	0	0	0	1	0	23	12	0	1	21	6	0
AUT7	0	1	0	7	11	4	0	2	12	0	0	1	0	0	0	24	2	0	0	23	15	8
AUT8	0	0	0	0	10	1	0	7	0	0	0	0	0	0	0	5	1	0	0	2	3	1
AUT9	0	0	na	na	na	na	na	na	na	na	na	0	0	na	na	na	na	na	na	na	na	na
AUT10	0	0	0	10	0	10	0	12	0	0	0	0	0	0	1	0	4	0	0	9	7	1
AUT11	0	0	0	6	1	0	0	1	0	1	0	0	0	0	0	6	0	1	0	1	0	0
AUT12	0	0	0	10	0	1	0	9	0	0	0	0	0	0	0	9	9	12	0	6	0	1
AUT13	0	0	0	0	0	0	0	0	0	0	0	1	0	0	0	5	6	0	0	6	0	2
AUT14	0	0	0	0	0	0	0	0	0	0	0	0	22	0	6	0	1	0	9	11	2	1
AUT15	0	0	0	0	1	0	0	12	12	0	0	3	4	0	0	21	0	2	1	4	0	0
HUN1	0	0	0	12	0	0	0	12	0	0	0	10	1	3	0	11	9	0	0	12	0	13
HUN2	0	0	0	0	0	0	0	0	0	0	0	6	7	0	1	25	13	0	0	21	10	0
HUN3	0	0	0	12	0	0	0	11	12	0	0	0	0	0	0	4	2	0	0	0	1	0
HUN4	0	0	0	12	0	na	0	12	0	0	0	12	0	0	3	27	na	7	6	32	8	12
HUN5	0	0	0	12	0	0	0	0	12	0	0	3	0	0	1	0	0	0	0	0	0	0
HUN6	0	0	0	0	0	0	0	0	0	0	0	2	0	0	0	0	0	0	0	1	0	1
HUN7	0	0	0	4	0	na	0	12	0	na	na	1	3	0	3	4	na	0	0	27	na	na
HUN8	0	0	na	na	na	na	na	na	12	na	na	11	1	na	na	na	na	na	na	0	na	na
HUN9	0	0	0	5	0	0	0	0	12	0	0	3	0	3	1	4	0	0	0	0	10	0
HUN10	0	0	0	1	0	0	0	0	0	0	0	1	0	0	0	1	0	0	0	0	4	0
HUN11	0	0	na	na	na	na	na	na	12	0	0	10	0	na	na	na	na	na	na	19	12	12
HUN12	0	0	0	6	1	0	0	10	10	0	0	11	0	1	0	6	0	0	0	1	0	0
HUN13	0	0	0	0	0	0	0	9	0	0	0	18	6	2	7	9	0	3	7	27	24	21
HUN14	0	0	0	7	0	na	0	12	11	0	0	15	7	0	5	23	na	9	18	26	4	0
HUN15	0	0	0	5	0	0	0	0	6	3	0	0	0	0	0	0	0	0	0	0	0	1

Table 3 Amphibian taxa identified through eDNA metabarcoding.

Name	Scientific name
European fire-bellied toad	*Bombina bombina*
Common toad	*Bufo bufo*
European green toad	*Bufotes viridis*
European tree frog	*Hyla arborea*
Smooth newt	*Lissotriton vulgaris*
Water frogs	*Pelophylax* spp.
Common spadefoot	*Pelobates fuscus*
Agile frog	*Rana dalmatina*
Common frog	*Rana temporaria*
Danube crested newt	*Triturus dobrogicus*

Table 4 A summary of the mean, SD, minimum and maximum values of the environmental variables.

Environmental variables	Code	Mean	SD	Min	Max
Lateral hydrological connectivity (%)	LHC	37.626	36.850	0.000	100.000
Area of the waterbody (km^2)	AREA	0.910	2.981	0.003	15.776
Depth of the waterbody (m)	DEPTH	1.003	0.962	0.025	4.622
Velocity (cm/s)	VELOCITY	28.310	51.203	0.000	220.000
Trees and large bushes bank vegetation (%)	WOODY	46.259	29.480	0.000	100.000
Herbaceous bank vegetation (%)	HERBACEOUS	51.845	30.540	0.000	100.000
Rip-rap and concrete bank cover (%)	ARTIFICIAL	1.897	5.888	0.000	30.000
Emergent vegetation (%)	EMVEG	9.164	17.740	0.000	90.000
Submerged vegetation (%)	SUBVEG	9.784	12.509	0.000	45.000
Floating vegetation (%)	FLOATVEG	4.845	8.460	0.000	26.500
Floating algae (%)	ALGAE	2.526	5.675	0.000	21.250
Open water (%)	OPENWATER	69.716	24.321	10.000	100.000
Water temperature (°C)	WTEMP	19.248	2.800	9.800	23.675
pH	PH	7.793	0.542	6.740	8.970
Dissolved oxygen concentration (mg/L)	OXY	9.774	2.197	4.210	12.998
Conductivity (µS/cm)	COND	475.905	195.958	303.250	1059.500
Total suspended solids (mg/L)	TSS	15.632	9.327	2.968	41.924
Total dissolved phosphorus concentration (µg/L)	TP	98.449	95.175	16.778	318.554
Chromophoric dissolved organic matter (Pt colour mg/L)	CDOM	28.053	20.953	3.616	82.850
Chlorophyll-a concentration (µg/L)	CHLO-A	25.697	31.642	0.214	104.070

Table 5 The results and principal component scores for the local habitat structure predictor group. Coefficients that are both bold and underlined indicate moderate to strong correlations (|r| > 0.3). For a description of the variables see Table S2.

Variable	PC1	PC2	PC3	PC4	PC5	PC6
AREA	**-0.51**	-0.21	-0.22	**-0.36**	**0.72**	0.00
DEPTH	**-0.44**	0.06	**0.87**	0.18	0.06	0.00
VELOCITY	**-0.39**	**-0.36**	**-0.33**	**0.78**	-0.10	0.00
WOODY	-0.21	**0.66**	-0.18	0.12	0.04	**0.69**
HERBACEOUS	**0.30**	**-0.60**	0.19	-0.03	0.09	**0.71**
ARTIFICIAL	**-0.51**	-0.20	-0.10	**-0.47**	**-0.67**	0.14
**Proportion variance explained**	0.54	0.32	0.07	0.06	0.01	0.00

Table 6 The results and principal component scores for vegetation predictor group. Coefficients that are both bold and underlined indicate moderate to strong correlations (|r| > 0.3). For a description of the variables see Table S2.

Variable	PC1	PC2	PC3	PC4	PC5
EMVEG	**0.52**	-0.24	**0.59**	0.21	**-0.52**
SUBVEG	**0.38**	**0.38**	**-0.65**	**0.37**	**-0.38**
FLOATVEG	0.02	**0.75**	0.27	**-0.55**	-0.24
ALGAE	**0.33**	**-0.46**	**-0.37**	**-0.72**	-0.18
OPENWATER	**-0.69**	-0.17	-0.09	0.01	**-0.70**
**Proportion variance explained**	0.39	0.24	0.19	0.15	0.01

Table 7 The results and principal component scores for physical and chemical predictor group. Coefficients that are both bold and underlined indicate moderate to strong correlations (|r| > 0.3). For a description of the variables see Table S2.

Variable	PC1	PC2	PC3	PC4	PC5	PC6	PC7	PC8
WTEMP	0.07	**0.56**	-0.07	**0.75**	-0.05	-0.26	-0.19	-0.08
PH	**-0.39**	0.11	0.01	0.21	**0.74**	**0.32**	**0.38**	0.00
OXY	**-0.33**	**-0.35**	**0.52**	0.24	-0.17	**-0.51**	**0.38**	-0.08
COND	-0.05	**-0.46**	**-0.70**	**0.37**	-0.24	0.11	0.28	-0.05
TSS	0.27	**-0.56**	0.27	**0.38**	0.30	0.16	**-0.51**	0.15
TP	**0.50**	-0.08	-0.03	-0.08	0.29	-0.19	0.17	**-0.77**
CDOM	**0.49**	0.02	-0.14	-0.04	0.28	**-0.42**	**0.34**	**0.61**
CHLO-A	**0.41**	0.11	**0.38**	0.21	**-0.33**	**0.57**	**0.44**	0.06
**Proportion variance explained**	0.42	0.16	0.13	0.12	0.08	0.04	0.03	0.01

Table 8 Summary results of the final linear models following forward selection used to identify the PCNM axes for the relative abundance of *Pelophylax* spp. and *B. bufo*, LHC, VEG, STRUCT and PHYSCHEM, separately. Significant PCNM axes are highlighted in bold, while marginally significant results are indicated in italics. For a description of the variables see Table S2.

Model	Predictor	Estimate	SD	t	p
Pelophylax complex	PCNM1	3.934	6.322	0.622	0.540
	PCNM2	6.970	6.322	1.103	0.282
	PCNM3	-1.418	6.322	-0.223	0.836
	PCNM4	2.499	6.322	0.394	0.708
	PCNM5	-6.334	6.322	-1.002	0.337
	PCNM6	0.356	6.322	0.065	0.967
Bufo bufo	PCNM1	2.660	3.525	0.755	0.458
	PCNM2	1.080	3.525	0.306	0.762
	PCNM3	-4.465	3.525	-1.267	0.219
	PCNM4	-2.069	3.525	-0.587	0.563
	PCNM5	*-6.655*	*3.368*	*-1.976*	*0.058*
	PCNM6	0.658	3.525	0.187	0.854
LHC	PCNM1	-12.075	38.652	-0.312	0.758
	PCNM2	-20.945	38.652	-0.542	0.593
	PCNM3	-44.549	38.652	-1.153	0.261
	PCNM4	-21.329	38.652	-0.552	0.587
	PCNM5	45.705	38.652	1.182	0.250
	PCNM6	6.493	38.652	0.168	0.868
VEG	PCNM1	-1.989	1.408	-1.412	0.172
	PCNM2	1.626	1.408	1.155	0.261
	PCNM3	1.750	1.408	1.243	0.227
	PCNM4	0.757	1.408	0.538	0.596
	PCNM5	-0.971	1.408	-0.689	0.498
	PCNM6	0.244	1.408	0.173	0.864
STRUCT	PCNM1	0.187	1.947	0.096	0.924
	PCNM2	-0.458	1.947	-0.235	0.816
	PCNM3	-0.104	1.947	-0.053	0.958
	PCNM4	0.275	1.947	0.141	0.889
	PCNM5	-2.627	1.947	-1.349	0.191
	PCNM6	-0.742	1.947	-0.381	0.707
PHYSCHEM	**PCNM1**	**6.826**	**1.214**	**5.625**	**<0.001**
	PCNM2	0.197	1.232	0.160	0.874
	PCNM3	1.495	1.232	1.214	0.238
	PCNM4	*2.293*	*1.214*	*1.890*	*0.071*
	**PCNM5**	**-2.546**	**1.214**	**-2.098**	**0.046**
	PCNM6	1.070	1.232	0.868	0.395

Table 9 Direct effects of the structural equation model for the relative abundance of *Pelophylax* spp. are provided showing standardized and unstandardized coefficients, along with their standard errors, z-values and p-values for each direct effect. Significant regressions are highlighted in bold, whereas marginally significant results are shown in italics.

**Response Variable**	**Predictor Variable**	**Standardized coefficient**	**Unstandardized coefficient**	**SE**	**Z**	**P**
PHYSCHEM	**PCNM1**	**0.680**	**1.254**	**0.186**	**6.757**	**<0.001**
	**PCNM4**	**0.200**	**0.369**	**0.186**	**1.981**	**0.048**
	PCNM5	*-0.186*	*-0.344*	*0.191*	*-1.803*	*0.071*
	**LHC**	**-0.319**	**-0.588**	**0.192**	**-3.060**	**0.002**
VEG	**LHC**	**-0.609**	**-0.853**	**0.206**	**-4.138**	**<0.001**
STRUCT	**LHC**	**-0.545**	**-0.984**	**0.281**	**-3.504**	**<0.001**
Pelophylax complex	**PHYSCHEM**	**0.393**	**0.214**	**0.087**	**2.453**	**0.014**
	**VEG**	**0.384**	**0.276**	**0.131**	**2.102**	**0.036**
	STRUCT	0.171	0.095	0.096	0.991	0.322
	LHC	0.107	0.107	0.218	0.492	0.623

Table 10 The standardized and unstandardized indirect and total effects of lateral hydrological connectivity (LHC) through PHYSCHEM. VEG and STRUCT mediators and the overall indirect and total effects of LHC on the relative abundance of *Pelophylax* spp. Significant regressions are highlighted in bold, whereas marginally significant results are shown in italics.

**Pathway**	**Standardized coefficient**	**Unstandardized coefficient**	**SE**	**Z**	**P**
Indirect effects					
PHYSCHEM	*-0.125*	*-0.126*	*0.066*	*-1.914*	*0.056*
VEG	*-0.234*	*-0.235*	*0.126*	*-1.874*	*0.061*
STURCT	-0.093	-0.094	0.099	-0.954	0.340
Total effects					
PHYSCHEM	-0.019	-0.019	0.211	-0.088	0.930
VEG	-0.127	-0.128	0.195	-0.655	0.512
STURCT	0.013	0.013	0.198	0.067	0.947
Overall indirect effect	**-0.453**	**-0.455**	**0.173**	**-2.637**	**0.008**
Total effect	**-0.346**	**-0.348**	**0.165**	**-2.111**	**0.035**

Table 11 Direct effects of the structural equation model for the relative abundance of *B. bufo* are provided, showing standardized and unstandardized coefficients, along with their standard errors, z-values, and p-values for each direct effect. Significant regressions are highlighted in bold, whereas marginally significant results are shown in italics.

**Response Variable**	**Predictor Variable**	**Standardized coefficient**	**Unstandardized coefficient**	**SE**	**Z**	**P**
PHYSCHEM	**PCNM1**	**0.680**	**1.254**	**0.186**	**6.757**	**<0.001**
	**PCNM4**	**0.200**	**0.369**	**0.186**	**1.981**	**0.048**
	PCNM5	*-0.186*	*-0.344*	*0.191*	*-1.803*	*0.071*
	**LHC**	**-0.319**	**-0.588**	**0.192**	**-3.060**	**0.002**
VEG	**LHC**	**-0.609**	**-0.853**	**0.206**	**-4.138**	**<0.001**
STRUCT	**LHC**	**-0.545**	**-0.984**	**0.281**	**-3.504**	**<0.001**
Bufo bufo	PHYSCHEM	0.146	0.078	0.092	0.850	0.395
	VEG	0.307	0.216	0.135	1.593	0.111
	**STRUCT**	**0.407**	**0.222**	**0.099**	**2.232**	**0.026**
	LHC	0.262	0.258	0.226	1.143	0.253
	PCNM5	-0.233	-0.229	0.157	-1.456	0.145

Table 12 The standardized and unstandardized indirect and total effects of lateral hydrological connectivity (LHC) through PHYSCHEM, VEG and STRUCT mediators and the overall indirect and total effects of LHC on the relative abundance of *B. bufo*. Significant regressions are highlighted in bold, whereas marginally significant results are shown in italics.

**Pathway**	**Standardized coefficient**	**Unstandardized coefficient**	**SE**	**Z**	**P**
Indirect effects					
PHYSCHEM	-0.047	-0.046	0.056	-0.819	0.413
VEG	-0.187	-0.184	0.124	-1.487	0.137
STURCT	*-0.222*	*-0.218*	*0.116*	*-1.883*	*0.060*
Total effects					
PHYSCHEM	0.216	0.212	0.217	0.979	0.328
VEG	0.076	0.074	0.199	0.373	0.709
STURCT	0.041	0.040	0.213	0.188	0.851
Overall indirect effect	**-0.455**	**-0.448**	**0.179**	**-2.509**	**0.012**
Total effect	-0.193	-0.190	0.173	-1.093	0.274
